# Allogeneic mesenchymal stem cells for treatment of severe burn injury

**DOI:** 10.1186/s13287-019-1465-9

**Published:** 2019-11-21

**Authors:** Marc G. Jeschke, Sarah Rehou, Matthew R. McCann, Shahriar Shahrokhi

**Affiliations:** 1 Sunnybrook Research Institute, Toronto, Ontario Canada; 2 Ross Tilley Burn Centre, Sunnybrook Health Sciences Centre, 2075 Bayview Ave. D7 04, Toronto, Ontario M4N 3M5 Canada; 3 Division of Plastic and Reconstructive Surgery, Department of Surgery, Faculty of Medicine, University of Toronto, Toronto, Ontario Canada; 4 Department of Immunology, Faculty of Medicine, University of Toronto, Toronto, Ontario Canada; 5 Institute of Medical Science, Faculty of Medicine, University of Toronto, Toronto, Ontario Canada

**Keywords:** Burns, Cell therapy, Mesenchymal stem cells, Wound healing

## Abstract

The most important determinant of survival post-burn injury is wound healing. For decades, allogeneic mesenchymal stem cells (MSCs) have been suggested as a potential treatment for severe burn injuries. This report describes a patient with a severe burn injury whose wounds did not heal with over 18 months of conventional burn care. When treated with allogeneic MSCs, wound healing accelerated with no adverse treatment complications. Wound sites showed no evidence of keloids or hypertrophic formation during a 6-year follow-up period. This therapeutic use of allogeneic MSCs for large non-healing burn wounds was deemed safe and effective and has great treatment potential.

## Introduction

In addition to substantial morbidity and mortality during the acute phase of critical illness, burn injury can also lead to post-injury scar tissue formation with long-term functional and psychosocial consequences [1]. The use of stem cells in the treatment of burns remains challenging and continues to be an area under intense research [2, 3]. In 1984, the *New England Journal of Medicine* reported the first use of autologous cultured human epithelium to treat two pediatric patients with > 97% total body surface area (TBSA) involvement, where autologous full-thickness biopsies were cultured and single-cell suspensions were expanded to provide coverage [4]. This provided evidence that isolated biopsies contain cells that when cultured can preserve the potential to initiate regenerative processes upon transplantation to the wound site. Recent efforts have focused on identifying ideal cell sources with these mitigating capabilities.

Currently, human umbilical cord and amniotic membrane MSCs (mesenchymal stem cells) are being explored for potential therapeutic use. These sources are considered non-invasive, low cost, and abundant. The MSCs from them can be easily grown and maintained in tissue culture [5]. Once administered to the donor bed, these cells are able to migrate to the source of tissue damage to support endogenous stem cells. Additionally, their immunosuppressive properties allow them to withstand acute cellular rejection [5]. Trials completed to date have not demonstrated long-term adverse effects, such as neoplasm formation or cellular rejection with the use of MSCs [6].

The amniotic membrane is the innermost fetal membrane layer of the amniotic sac which protects the fetus. It also provides a physiologically and immunologically distinct environment for the fetus to thrive [7]. In 1912, Stern and Sabella conducted pioneer work on amniotic membranes where they successfully used freshly isolated amniotic tissue to treat a relatively small burn (9 square inches) [8]. This pivotal work paved way for clinical use of MSCs. When removing the dressing from patients’ after 2 days, Stern documented the presence of distinct membrane layers [8]. Perhaps unknowingly, Stern had identified that MSCs were able to adapt and integrate with the wound while the chorionic layer contained epithelial cells that could be removed.

While the application of amniotic MSCs in burn care has fluctuated throughout the last century, there was a resurgence in their use in the 1990s. Since then, we have developed a better understanding of the amnion’s immunoregulatory, antimicrobial, anti-fibrotic, and anti-scarring characteristics [9]. These characteristics can improve wound healing in patients with severe burns [10]. However, treatment of full-thickness burns with the amniotic membrane continues to be challenging with the risk of microbial contamination and infection [11]. To overcome the potential for contamination and optimize cellular expansion, we opted for experimental therapy with MSCs in a patient with severe full-thickness burn injuries.

## Case report

### Local hospital

**Fig. 1:**
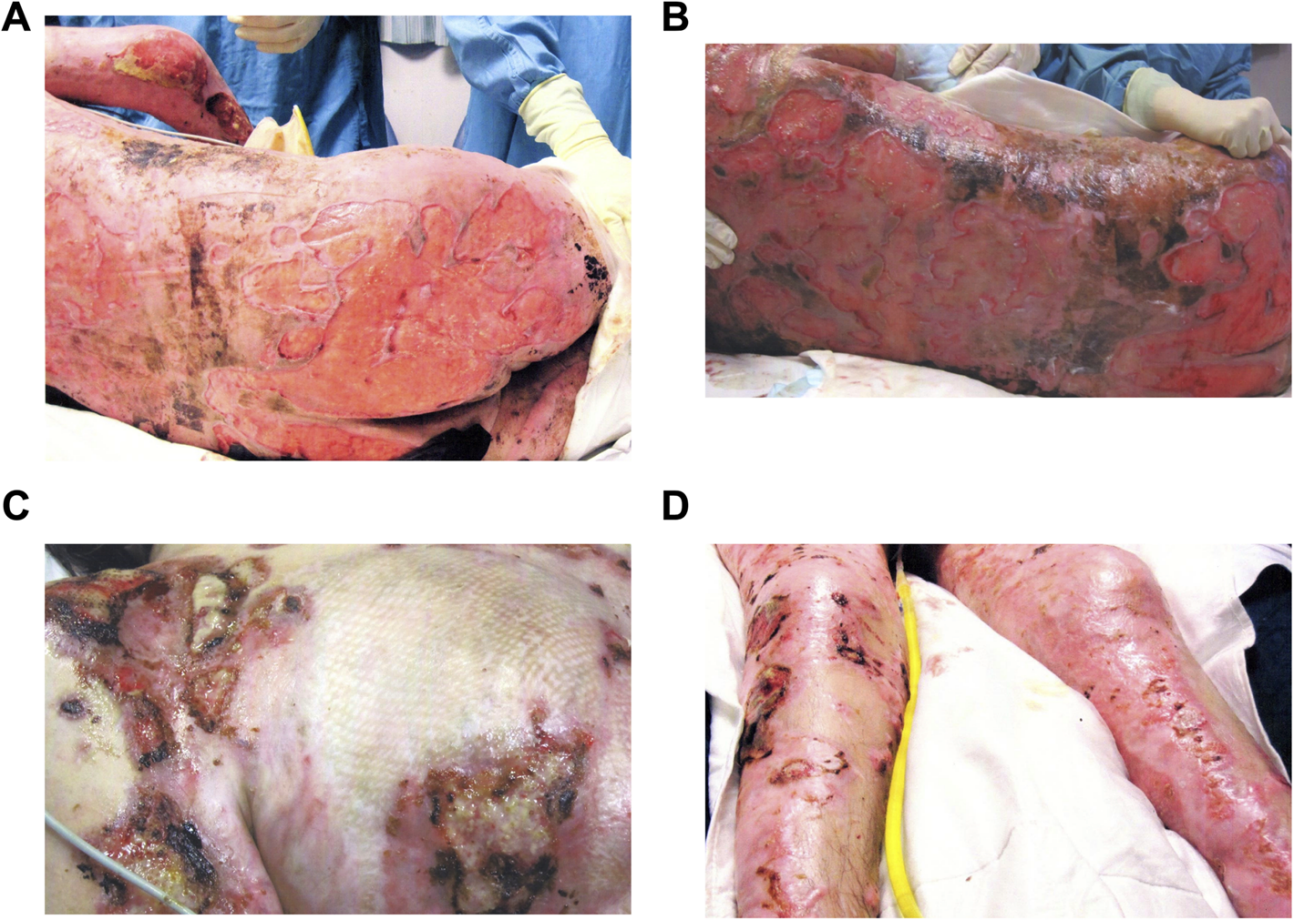
Open and infected wounds 18 months after burn injury. Posterior trunk, 72 weeks post-injury (**a**); posterior trunk, 73 weeks post-injury (**b**); anterior trunk, 73 weeks post-injury (**c**); anterior lower extremities, 73 weeks post-injury (**d**)

A male in his mid-twenties was admitted to a local, tertiary care center with > = 70% TBSA burns, mostly full thickness, and had also sustained smoke inhalation injury as a result of a house fire. The areas involved included the chest, back, bilateral arms, hands, thighs, feet, and buttocks. The patient’s initial surgeries included escharotomy of his right forearm and a transmetatarsal amputation of the right and left foot. He had in total 13 surgeries for excision and application of skin grafts, a tracheostomy, an ileostomy for fecal diversion due to non-healing buttock wounds, and a percutaneous gastrostomy tube placement. During this time, the patient developed chronic infections with multi-drug resistant *Pseudomonas.* His pain management had also become increasingly complicated. Eighteen months post-injury, approximately of more than one third of his initial wounds remained open and were severely infected with a plethora of bacteria. At this point, the patient was transferred to a specialized burn center (Fig. 1).

### Provincial burn center

On admission to our burn center, initial assessment demonstrated wounds with significant hyper-granulation with profound bacterial colonization/infection. These wounds, including the back, chest, right arm, and bilateral legs, were subsequently debrided with application of allograft. Documented assessments of the sites over the next 2 weeks found that the allografting failed and that “the majority of the wounds remained open, and infected with minimal to no healing or epithelization, particularly the large regions of the back and buttocks”. The wounds were infected and contaminated with *Enterobacter cloacae*, *Enterococcus* species, *Klebsiella pneumoniae*, *Pseudomonas aeruginosa*, *Staphylococcus aureus*, and *Yeast*. The allograft application failed to result in vascularization, and subsequently, the wounds did not heal. Given the longstanding history of the wounds and multiple complications and failures, it was determined that conventional treatment would not result in a favorable outcome. Therefore, a novel treatment approach was needed in order to adequately heal these wounds. In due course, we sought approval from our institution to use amniotic and umbilical allogeneic stem cell transplantation from two donors. The hypothesis was that allogeneic MSCs could induce an immune response that would help to clear the infection. We also believed that these cells would stimulate the release of growth factors which would accelerate wound healing. In a later surgery, the hyper-granulated areas were excised and the cord-lining membrane mesenchymal stem cells (CL-MSCs) were applied topically using a fibrin sealant spray along the back and buttocks. Allografts were then applied over the area to temporary close and protect the wounds.

Three weeks postoperatively, the attending staff surgeon determined that the patient’s open wound decreased to approximately half of the original one third and showed significantly decreased infection. At this stage, another surgery was undertaken, at which time, commercially produced MSCs were subcutaneously injected into the hyper-granulated tissue. One week after the second MSC application, the patient’s open burn wounds decreased to about one seventh. A final surgery involved injecting the wound bed with 20 cm^3^ of platelet-rich plasma and application of a negative-pressure wound therapy device.

Two months after the second MSCs treatment, less than 3% of wounds remained open, at which point a final surgery was performed in order to autograft from the remaining wound using the patient’s scalp skin as donor. Five months after admission to our burn center and four and a half months since the initial MSCs treatment, the patient was discharged for rehabilitation care with no open wounds.

### Long-term assessment

Nineteen months post-discharge from our burn center, the patient returned for contracture release of his right axilla and hip. At this time, we found no evidence of hypertrophic scars, keloids, or wound breakdown. Scar contracture releases with transposition flaps were undertaken on the right axilla and hip. During this time, the patient underwent right elbow ulnar nerve neurolysis, excision of heterotopic ossification, and soft tissue contracture release.

**Fig. 2:**
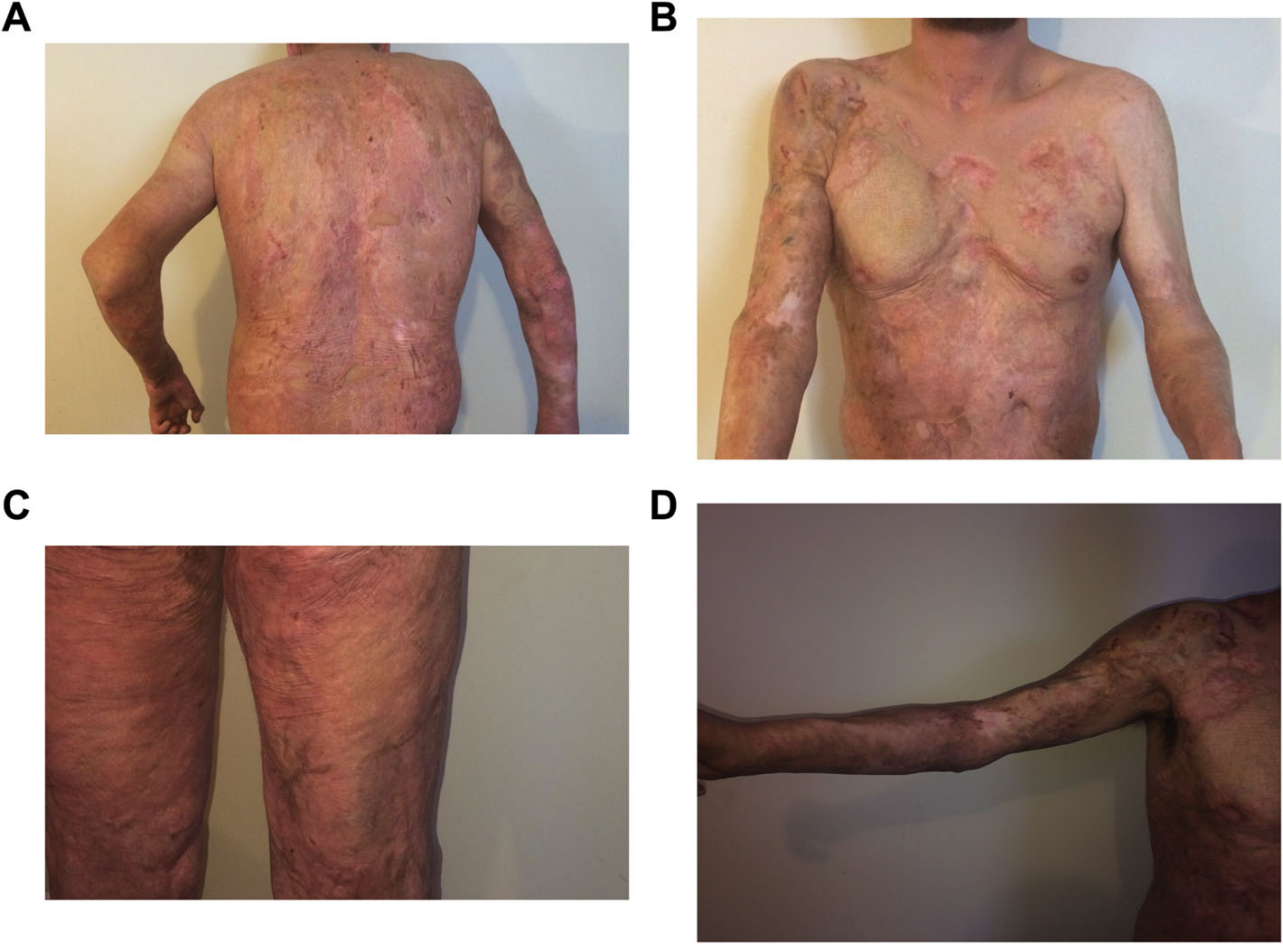
Six years after mesenchymal stem cell application. Posterior trunk (**a**), anterior trunk (**b**), posterior lower extremities (**c**), right upper extremity (**d**)

At a 6-year follow-up, the patient was in excellent health with substantial amounts of complete epithelialization, particularly on the back, buttocks, and bilateral legs. Importantly, there was minimal hyperpigmentation and hypertrophic scarring as well as no evidence of keloids following MSC administration (Fig. 2). The patient maintained excellent ranges of motion and transitioned well into daily activities of living.

## Methods

### Ethics

Treatment: This patient was profoundly ill and we contacted our institutional Research Ethics Board and the Chief Medical Executive to ask for compassionate use approval. Compassionate use approval was granted, and subsequently, we obtained consent from the substitute decision maker for treatment. The patient was assented at a later time during his hospital stay. Case report: A case report does not require approval from our institutional Research Ethics Board. Consent was obtained from the patient to allow their information and images to be published.

### Isolation, expansion, and culture of MSCs

Our initial seeding of CL-MSCs into the burn wounds was conducted using human umbilical cord lining membrane (subamniotic) MSCs isolated using a previously established protocol in our lab [12]. Briefly, human umbilical cord samples from healthy donors were screened, dissected at the envelope membranes, and cultured. These distinct CL-MSCs have membrane expression of CD73 and CD105, MSC markers, and Oct-4, Nanog, and SSEA-4, preserved stem cell markers Oct-4. However, they lack Sox-2, FGF-4, TERT, and Rex-1markers found on other embryonic stem cells. Cultures were tested for microbiological contamination before being prepared for clinical application.

### Initial MSC seeding

After the hyper-granulated tissue was excised and cleaned, CL-MSCs were placed in Ringer’s lactate and mixed with fibrin sealant (3 million cells/mL). Four milliliters of the stem cell/sealant cocktail was topically applied to the back, buttocks, and legs. The wounds were allografted for protection, covered using paraffin gauze and antimicrobial dressings, and finished with bolster dressings.

### Secondary MSC injections

We next used Ovation® MSCs derived from the chorion layer of donor placentas from Osiris Therapeutics, Inc. (Columbia, MD, USA) [13]. Ovation® MSCs were commercially available at the time. However, Osiris Therapeutics, Inc., stopped manufacturing the product in 2014. Twelve vials of MSCs were thawed and individually placed in Ringer’s lactate solution before being injected under the granulated tissue in the patient’s back, buttocks, and legs. Injections were conducted homogenously throughout the open wound regions and were systematically placed near the perimeter of the wounds to stimulate re-epithelization. Once more, the wounds were protected with allograft and subsequent dressings were applied.

## Discussion

In this report, we describe the use of stem cell therapy on a male patient in his mid-twenties with a 70% TBSA burn injury. Eighteen months prior to admission to the burn center, conventional means of burn treatment left this patient with multiple in-hospital complications and in an overall poor condition. With the administration of allogeneic MSCs, we were able to reduce open wounds from one third to less than 3% and significantly heal infections in a short time period. However, we were concerned about the possibility of long-term keloids and/or hypertrophic scarring with therapeutic use of MSCs. We observed the patient for over 6 years and found the patient had an excellent wound recovery trajectory, with limited scarring relative to similar burn injuries and no adverse side effects, such as neoplastic formation or adverse immune response. The lack of severe hypertrophic scarring is particularity remarkable considering the amount of time the wounds had remained open and infected. This is even more surprising since most of his wounds were full-thickness, which are generally slow to heal and have a greater risk for developing pathological, hypertrophic scars [14].

While the use of MSCs in burn wounds treatment has advanced in recent years, most reported trials describe patients with < 80% TBSA of which, only approximately 30% are full-thickness burns [15]. Most are partial and deep-partial thickness burns with minimal %TBSA [16]. In contrast, this patient was admitted with a large surface area (70% TBSA) burn, mostly full-thickness, as well as an inhalation injury with a resultant mortality risk of 80% based on the modified Beaux score [17]. Despite the observed recovery, the mechanisms by which MSCs contribute to wound healing in patients with large, severe burns remain unknown.

It has been postulated that MSCs serve dual functions. They are thought to first release mediators that influence inflammation and stimulate angiogenesis and then differentiate into multilayered epidermal-like structures that aid in wound closure [18]. In a seminal paper by Wu et al.*,* it was shown that differentiated bone marrow-derived MSCs released VEGF-α and Ang-1 which stimulated endothelial cell proliferation and subsequent tissue migration, ultimately increasing angiogenesis [19]. In rodent burn models, human-umbilical MSCs modulated expression at the cellular level, specifically increased VEGF expression upstream of wound site microvessel formation, increased anti-inflammatory mediators IL-10 and TSG-6, and decreased proinflammatory cytokines IL-1, IL-6, and TNF-α [20]. It is possible that exosome release may account for some of the anti-inflammatory effects observed. Exosomes contain miR-181c which act to suppress toll-like receptor 4 expression and may lead to reduced downstream pro-inflammatory factors, TNF-α and IL-1β [21].

Additionally, the use of either the amniotic membrane or CL-MSCs might have different results. The use of the amniotic membrane in pediatric patients with partial-thickness facial burns did not significantly improve healing time, length of hospital stay, or development of hypertrophic scars [22]. In addition, dehydrated and irradiated amniotic membranes have shown little promise [23]. Since the use of MSCs in the clinical settings, specifically in wound care, has not yet been fully elucidated, we elected the use of two distinct methods: (1) a fibrin sealant-based topical application and (2) direct tissue injections. The first method followed by a dressing has been shown to be both safe and effective in applying MSCs and allowing them to persist and integrate within the wound area [24]. Evidence also shows that local injections of MSCs to and around the wound bed have been shown to accelerate wound healing in radiation burn treatments [25]. We hypothesize that the two treatments served separate functions in repairing the damaged tissue; the initial topical application served to quell inflammation and stimulate angiogenesis while the injections allowed for appropriate re-epithelization of the wounds.

One of the most interesting aspects during the treatment period was how the application of MSCs resulted in a reduction of burn wound infection. After the initial MSC application macroscopically and microscopically, the burn wounds cleared allowing allograft to be placed and initiate wound healing. MSCs have been studied in terms of their anti-infective properties, and several studies demonstrated that MSCs exert strong anti-infective effects via various mechanisms. A recent study by Johnson et al. showed that MSCs interact with type I and type II macrophages phenotype most likely by secretion of cathelicidin. The authors concluded that therapy with activated MSCs might be an effective non-antimicrobial approach to treatment of chronic, drug-resistant infections [26]. Another study by Hutton et al. found that MSCs increase bacterial killing capacity and bacterial clearance. They also showed that MSCs secrete an antimicrobial peptide LL-37 [27]. The signal of increased bacterial killing and clearing was confirmed by Johnson et al. as they showed that Cramp, an antimicrobial peptide, was significantly increased with MSCs [26]. It therefore appears that MSCs have strong anti-infective properties which we observed in our case report as well.

This case provides evidence for rapid re-epithelization of open wounds with MSCs without long-term consequences of hypertrophic scar formation, which inhibit mobility and have negative psychological effects. This case also demonstrates the immense potential of MSCs in the acute phase of burn care. Future research should investigate the efficacy of MSC use in the severely burned patient on a large scale.

## Data Availability

Not applicable.

## Abbreviations

CL-MSC: Cord-lining membrane mesenchymal stem cell
MSC: Mesenchymal stem cell
TBSA: Total body surface area
